# Utility of Blood Flow/Resistance Index Ratio (Q_x_) as a Marker of Stenosis and Future Thrombotic Events in Native Arteriovenous Fistulas

**DOI:** 10.3389/fsurg.2020.604347

**Published:** 2021-01-25

**Authors:** Alessandro Colombo, Michele Provenzano, Laura Rivoli, Cinzia Donato, Marinella Capria, Giuseppe Leonardi, Salvatore Chiarella, Michele Andreucci, Giorgio Fuiano, Davide Bolignano, Giuseppe Coppolino

**Affiliations:** ^1^Dialysis Unit, Hospital of Crotone, Crotone, Italy; ^2^Renal Unit, “Magna Graecia” University, Catanzaro, Italy; ^3^Unit of Nephrology, Department of Internal Medicine, Chivasso Hospital, Turin, Italy; ^4^Renal Unit, “Pugliese-Ciaccio” Hospital of Catanzaro, Catanzaro, Italy

**Keywords:** blood flow, resistance index, stenosis, thrombosis, haemodialysis, Doppler ultrasound, arteriovenous fistula

## Abstract

**Objective:** The resistance index (RI) and the blood flow volume (Q_a_) are the most used Doppler ultrasound (DUS) parameters to identify the presence of stenosis in arteriovenous fistula (AVF). However, the reliability of these indexes is now matter of concern, particularly in predicting subsequent thrombosis. In this study, we aimed at testing the diagnostic capacity of the Q_a_/RI ratio (Q_x_) for the early identification of AVF stenosis and for thrombosis risk stratification.

**Methods:** From a multicentre source population of 336 HD patients, we identified 119 patients presenting at least one “alarm sign” for clinical suspicious of stenosis. Patients were therefore categorized by DUS as stenotic (*n* = 60) or not-stenotic (*n* = 59) and prospectively followed. Q_a_, RI, and Q_X_, together with various clinical and laboratory parameters, were recorded.

**Results:** Q_a_ and Q_x_ were significantly higher while RI was significantly lower in non-stenotic vs. stenotic patients (*p* < 0.001 for each comparison). At ROC analyses, Q_x_ had the best discriminatory power in identifying the presence of stenosis as compared to Q_a_ and RI (AUCs 0.976 vs. 0.953 and 0.804; *p* = 0.037 and *p* < 0.0001, respectively). During follow-up, we registered 30 thrombotic events with an incidence rate of 12.65 (95% CI 8.54–18.06) per 100 patients/year. In Cox-regression proportional hazard models, Q_x_ showed a better capacity to predict thrombosis occurrence as compared to Q_a_ (difference between c-indexes: 0.012; 95% CI 0.004–0.01).

**Conclusions:** In chronic haemodialysis patients, Q_x_ might represent a more reliable and valid indicator for the early identification of stenotic AVFs and for predicting the risk of following thrombosis.

## Introduction

Arteriovenous fistula (AVF) is generally acknowledged as the best vascular access option for chronic haemodialysis (1). However, the clinical management of complications, principally stenosis, remains a major challenge for clinicians with a substantial impact on health resources worldwide (2–4). Stenosis not infrequently preludes to thrombotic events (5). Fluctuating shear stress in the stenotic region leads to intimal injury with a subsequent cascade of pro-inflammatory cytokines and proliferation of smooth-muscle cells, myofibroblasts and extracellular matrix that result in neo-intimal hyperplasia and subsequent risk of thrombosis (6).

Doppler ultrasound (DUS) surveillance is a practical and non-invasive approach to identify critical stenosis at risk of forthcoming thrombosis (7). Among DUS parameters, AVF blood flow volume (Q_a_) is nowadays considered the best parameter to detect stenosis while the resistance index (RI) usually provides less reliable information (8). Stenosis progression overtime generates resistance to flow, thereby increasing RI and decreasing blood flow Q_a_ (9). However, despite the lumen reduction, a high flow rate or a normal resistance index could paradoxically be observed in early stenotic AVFs, hence masquerading a good access patency (10, 11). Better indicators are therefore needed for improving critical stenosis identification and for stratifying the risk of thrombosis.

In the present study we tested the performance of a new surrogate Doppler parameter (Q_x_), obtained as the Q_a_/RI ratio, to detect the presence of AVF stenosis. We then evaluated the reliability of this new index to predict thrombosis occurrence in the brief to mid-term.

## Patients and Methods

### Study Cohort

From January to December 2017, haemodialysis patients from four different Italian centers (“*Magna Graecia*” University and “*Pugliese-Ciaccio*” Hospital of Catanzaro, Crotone and Chivasso Hospitals) were screened for signs of AVF stenosis, such as difficult cannulation, thrombi aspiration during cannulation, increasing of pre-pump arterial and post-pump venous pressure, unfeasibility to reach adequate blood flow rate or elongation of haemostasis. Patients who were positive to at least one of these “alarm signs” underwent specific DUS surveillance (12). Patients were then categorized as stenotic or not-stenotic basing on recognized criteria: 2 major criteria [reduction of vessel size greater than 50% and a ratio between systolic peak velocity (SPV) in the stenotic region and SPV in pre-stenotic region major of 2 (>2)] and a supplementary finding like drop of the access flow below 500 ml/min or drop of Q_a_ > 25% as compared to the previous measurements in AVF with Q_a_ <1,000 ml/min or residual diameter <2 mm (13). Demographic variables such as age, gender, dialysis duration, AVF type and duration, previous interventions (endovascular or surgical), intra-access venous and arterial pressure and recirculation were recorded.

### Exclusion/Inclusion Criteria

Patients included had a native vascular access created at least 4 months prior to our assessment to guarantee an adequate maturation period and were usually cannulated with classical rope-ladder technique. All subjects were on regular treatment haemodialysis with a rhythm of 4-h sessions three times a week with dry-weight stable for at least 2 months before entering the study and had achieved a normotensive oedema-free state. Exclusion criteria were the presence or a recent history of bleeding, malignancy, liver, thyroid or infectious diseases, alterations in leucocyte count or formula and/or treatment with steroids or immunosuppressors. The local ethics committee approved the study, and fully informed consent was obtained from all participants.

### Procedure

Two different blinded experienced examiners performed an ultra-sonographic study of AVFs 1 h before dialysis. The patient was in sitting position with elbow pad to stabilize the upper limb. After physical examination, B-mode and color Doppler images were obtained from the feeding artery, arterial anastomosis and the outflow vein to characterize vascular access and identify the presence of stenosis. Patency and continuity of the artery and vein were assessed from the distal forearm to the upper arm. The following parameters were recorded for each included subject with a standardized protocol (14, 15):

– Blood Flow (Q_a_) in the brachial artery by measuring the vessel diameter (D) and the time average velocity (TAV) through the formula: Q_a_ (ml/minute) = TAV (cm/second) ^*^ D (cm) × 60.
– Resistive index (RI) calculated by the following formula RI = (A – B)/A, (A = Peak Systolic Velocity, B = End Diastolic Velocity).

Finally, the Q_x_ was computed as the Q_a_/RI ratio. For each parameter, the mean of three different measures was considered for the statistical analysis.

After the baseline assessment, patients were then prospectively followed until the occurrence of a thrombosis event or up to the established end of the follow-up period (30th January 2020). Thrombosis was diagnosed by physical exam as no thrill, bruit or pulse and confirmed by DUS with an absence of flow on pulse-wave and color Doppler.

### Statistical Analysis

The statistical analysis was performed using STATA version 14 (Stata Corp.College Station, TX, USA), the GraphPad Prism and Med Calc package for figure depiction. Continuous variables were presented as mean ± SD or median (IQ range) as appropriate. Categorical variables were presented as percentage (%). Differences between groups were tested using the unpaired *t*-test for normally distributed values and Kruskal–Wallis analysis followed by a *post-hoc* test the Dunn's test for nonparametric values. Receiver operating characteristics (ROC) analysis was employed to evaluate variable's (Q_a_, RI, and Q_x_) ability for classifying disease status, which is, presence or absence of AVF critical stenosis. Comparison between ROC curves was assessed by a non-parametric approach (16). To find the best cut-off value for identifying the presence of AVF stenosis, the Youden index (*J*) was computed (17).

With respect to the risk of thrombosis, two multivariable Cox proportional hazards models were built including Q_a_ and Q_x_, respectively. We used Cox analyses to estimate hazard ratio (HR) and 95% CI of AVF thrombosis over time. For the model building process, univariate analysis testing the association between the main clinical variables and the onset of thrombosis was assessed by means of logistic regression analysis. The variables with *p* < 0.150 at univariate analysis were selected and included in the first multivariable Cox proportional hazard model. Next, backward variable selection method with an elimination criterion of *p* < 0.10 was performed to fit the second Cox model (Model with Q_a_ or Q_x_). Such as stringent cut-off for variables inclusion was used in order to avoid model overfitting, due to the limited sample of the cohort. The final model was adjusted by: systolic blood pressure, Q_a_ or Q_x_ and AVF dysfunction.

To assess the performance of the two models (Q_a_ vs. Q_x_), we compared the discrimination ability of each model by calculating the c-index for Cox proportional hazard model. CI of difference between c-indexes was calculated by the bootstrap method; bootstrap CI was calculated with 1,000 replicates and using the percentile method (18).

## Results

### Patients' Flow

**Figure 1:**
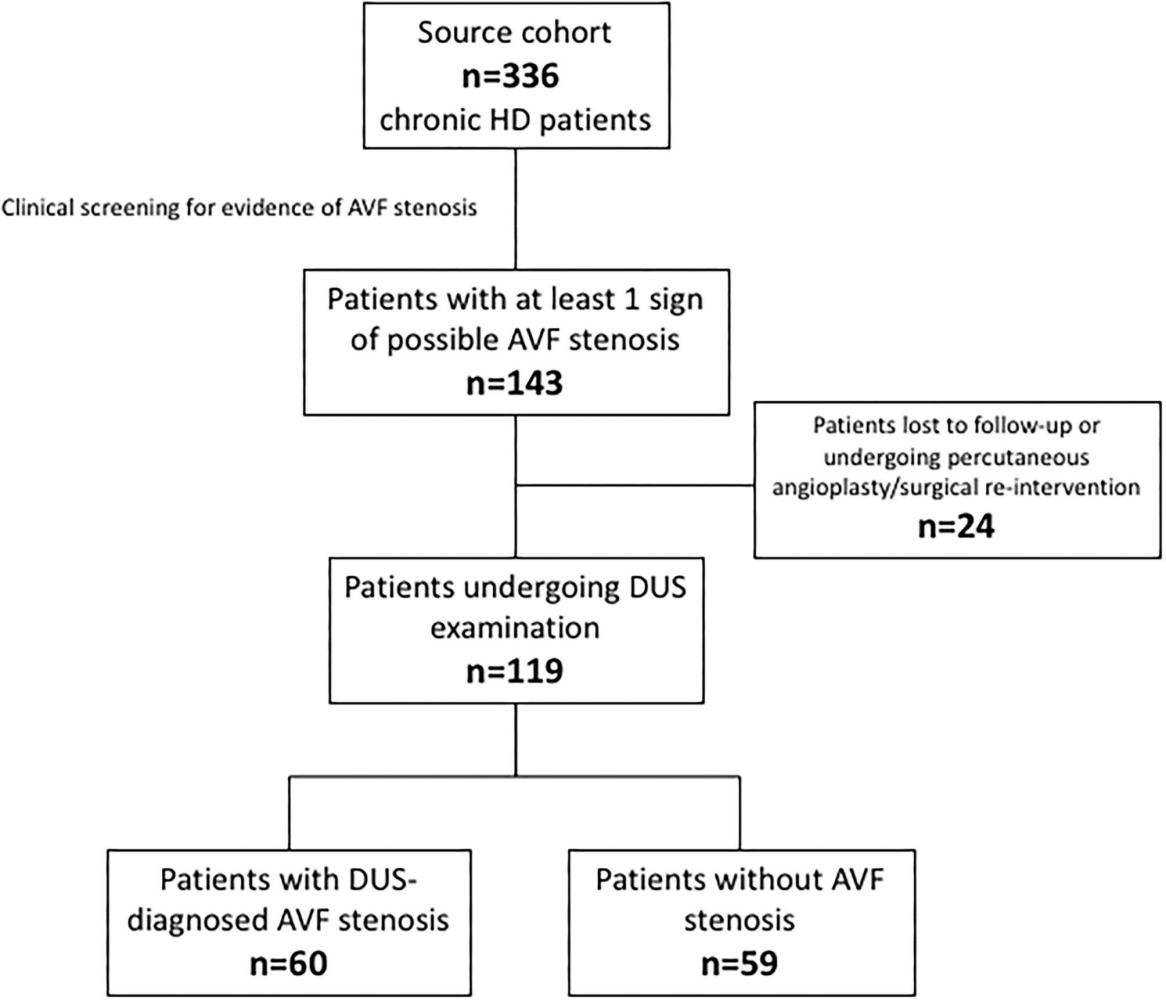
Patients' flow chart.

The source population consisted of 336 chronic haemodialysis patients. During the screening phase, 143 patients displayed at least one clinical sign of possible AVF stenosis. Twenty-four patients were excluded because lost at follow-up or because underwent a percutaneous angioplasty/surgical re-intervention before a thrombotic event. The final study cohort therefore consisted of 119 individuals who resulted almost equally distributed among the stenotic (*n* = 60) and non-stenotic (*n* = 59) group according to DUS examination. Figure 1 depicts the patients' flow.

### Baseline Assessment

The whole population included a moderate-high percentage of patients with type-II diabetes and CVD (29.4 and 58.0%, respectively). The principal types of AVF were distal (Radiocephalic fistulas) (63.0%) or proximal (Brachial artery–to–transposed basilic vein fistulas) (31.1%) with a marginal part represented by mid-arm (Brachiocephalic fistula) (5.9%) access. Overall Q_a_, RI, and Q_x_ were 1,437 (820–2,260) mL/min, 0.55 ± 0.09 and 2,727 (1,371–4,385) mL/min/RI, respectively.

The stenotic and non-stenotic groups were homogeneous for sex, CVD, smoking habit and laboratory parameters. Conversely, stenotic group showed a significant higher prevalence of diabetes, lower BMI, and higher frequency of previous access dysfunction as compared to non-stenotic group. Mean levels of Q_a_ and Q_x_ were significantly higher [2,255 (1,755–3,128) vs. 820 (663–1,304) mL/min and 4,377 (3,486–6,342) vs. 1,488 mL/min/RI (1,000–2,320)] while RI was significantly lower (0.49 ± 0.07 vs. 0.59 ± 0.08) (*p* < 0.001 for all comparisons) in not stenotic as compared to stenotic AVFs. Main baseline data and comparisons are summarized in Table 1.

**Table 1:** Demographic and clinical characteristics of patients at basal visit.

		**Stenosis**		
	**Overall**	**Yes**	**No**	***p***
Number (%)	119 (100)	60 (50.4)	59 (49.6)	–
Age (years)	62.7 ± 11.8	62.9 ± 12.3	62.6 ± 11.3	0.897
Males (%)	58.0	52.5	63.3	0.233
Diabetes (%)	29.4	37.3	21.7	0.061
History of CVD^*^ (%)	58.0	64.4	51.7	0.159
Smoking (%)	7.6	6.8	8.3	0.749
BMI (kg/m^2^)	26.3 ± 4.1	25.6 ± 3.0	27.0 ± 4.8	0.070
Systolic blood pressure (mmHg)	136 ± 19	137 ± 18	135 ± 20	0.538
Diastolic blood pressure (mmHg)	82 ± 8	82 ± 7	82 ± 9	0.770
Hemoglobin (g/dL)	10.8 ± 0.8	10.8 ± 0.8	10.8 ± 0.9	0.655
Haematocrit (%)	33 ± 4	33 ± 3	33 ± 3	0.734
Platelet (n^*^10∧3)	204 ± 52	206 ± 56	203 ± 48	0.799
Calcium (mg/dL)	9.4 ± 0.6	9.4 ± 0.5	9.4 ± 0.7	0.660
Phosphorus (mg/dL)	5.8 ± 1.2	5.90 ± 0.90	5.80 ± 1.42	0.639
Total cholesterol (mg/dL)	190 ± 40	192 ± 43	186 ± 38	0.412
Antihypertensive drugs (n)	1.5 ± 1	1.5 ± 1	1.5 ± 1	0.755
HD vintage (months)	30.0 ± 13.9	31.4 ± 14.4	28.7 ± 13.4	0.292
Vintage of AV access (years)^**^	26.2 ± 12.7	28.7 ± 13.5	23.8 ± 12.0	0.037
Types of AVF	–	–	–	0.003
Distal (Radiocephalic fistulas) (%)	63.0	78.0	48.3	
Mid-arm (Brachiocephalic fistulas) (%)	5.9	5.1	6.7	
Proximal (Brachial artery–to–transposed basilic vein fistulas) (%)	31.1	16.9	45.0	
Q_a_ (mL/min)	1,437 (820–2,260)	820 (663–1,304)	2,255 (1,755–3,128)	<0.001
RI	0.55 ± 0.09	0.59 ± 0.08	0.49 ± 0.07	<0.001
Q_x_ (mL/min/RI)	2,727 (1,371–4,385)	1,488 (1,000–2,320)	4,377 (3,486–6,342)	<0.001
History of access dysfunction (%)	26.9	39.0	15.0	0.003

* CVD, cardiovascular diseases: myocardial infarction, stroke, peripheral artery disease, chronic heart failure.

** *Dialysis vintage, length of time on dialysis*.

**Figure 2:**
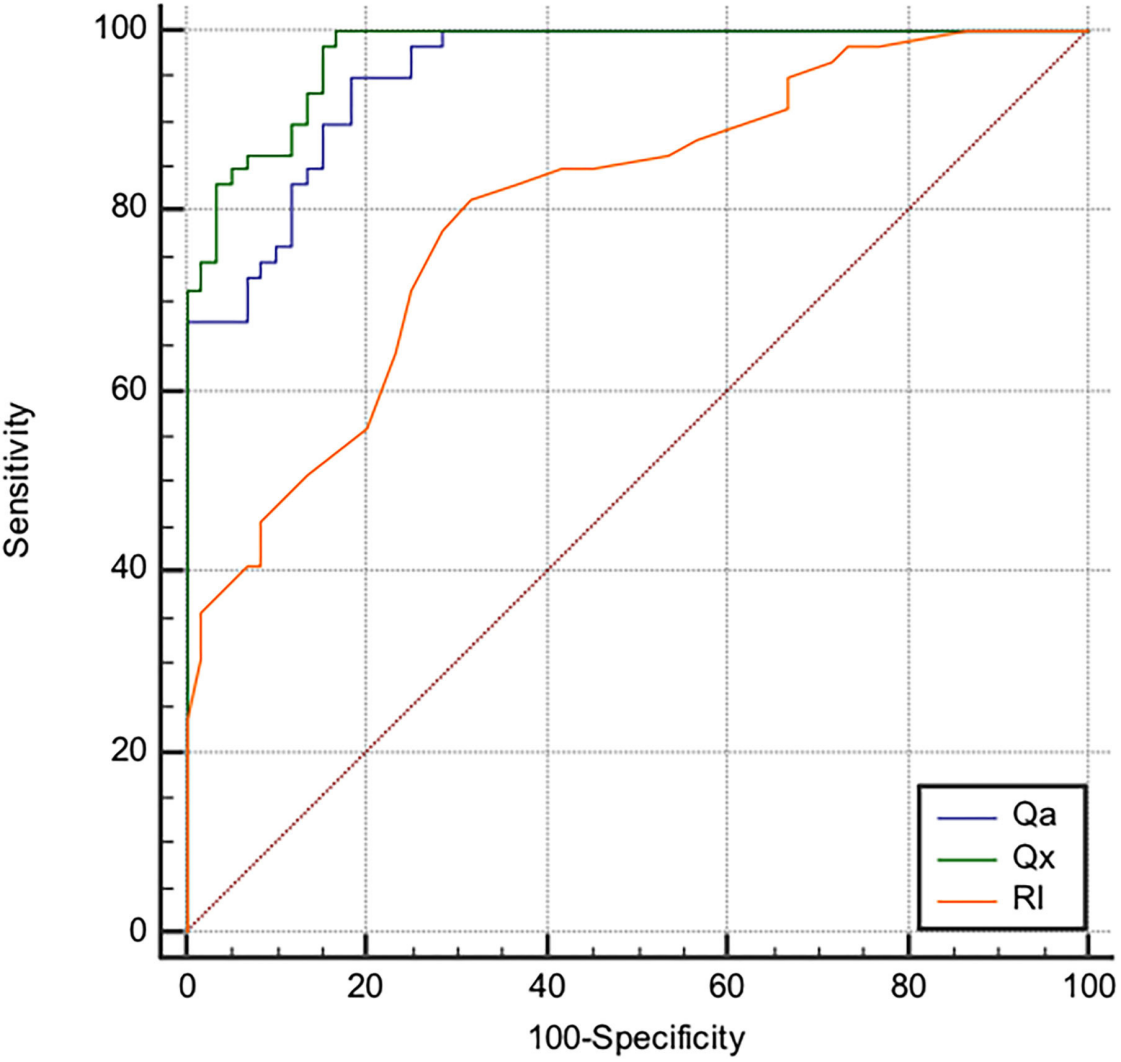
Receiver operating characteristics curves of AVF blood flow volume (Q_a_), resistance index (RI) and Q_a_/RI Resistance Index ratio (Q_x_) considering the presence of AVF stenosis as status variable. The area under the curve (AUC) for Q_a_, Q_x_, and RI were, respectively, 0.953, 0.976, and 0.804 (AUC Q_x_ vs. Q_a_ *p* = 0.037; AUC Q_x_ vs. RI *p* < 0.0001). The best cut-off values to identify the presence of stenosis were: 1,615 mL/min for Q_a_, with 94.9% (95% CI, 91.7–97.8) sensitivity and 81.7% (95% CI: 69.8–93.4) specificity; 3,333 mL/min/RI for Q_x_,with 99.9% (95% CI, 98.2–100) sensitivity and 83.3% (95%CI: 72.2–95.1) specificity; 0.53 for RI with 81.3% (95% CI, 69.1–90.3) sensitivity and 68.3% (95% CI 55–79.7) specificity.

ROC curves (Figure 2) showed that Q_x_ had a better discriminatory power in identifying the presence of AVF stenosis as compared to Q_a_ and RI. Values of Area Under the Curves (AUC) were 0.976, 0.953, and 0.804 for Q_x_, Q_a_, and RI, respectively, and the difference between curves was statistically significant (AUC Q_x_ vs. Q_a_ *p* = 0.037; AUC Q_x_ vs. RI *p* < 0.0001). When the cut-points *J* were derived from the ROC curves, the threshold of 3,333 mL/min/RI for Q_x_ (Sensitivity = 99.9%, Specificity = 83.3%) displayed the best accuracy in detecting the presence of stenosis as compared to that of Q_a_ (1,615 mL/min; Sensitivity = 94.9%, Specificity = 81.7%) and RI (0.53; Sensitivity = 81.3%, Specificity = 68.3%).

### Prospective Phase and Analyses

During a median follow-up of 24.5 (21.7–26.9) months, we registered 30 thrombotic events with an incidence rate of 12.65 (95% CI 8.54–18.06) per 100 patients/year. From the univariate linear regression analysis (Table 2), covariates with *p* < 0.150 (presence of stenosis, Q_a_ or Q_x_, RI, AVF dysfunction and systolic blood pressure) were included in the multivariate linear regression model. After backward selection of variables, with an elimination criterion of *p* < 0.100, systolic blood pressure, the presence of AVF dysfunction, low Q_a_ and Q_x_ were independently and significantly associated to the development of thrombosis over time (Table 3).

**Table 2:** Univariate logistic regression analysis for the onset of thrombosis.

**Characteristics**	**β Coefficient (95% CI)**	***p***
Age, *years*	0.007 (−0.029–0.042)	0.708
**Gender**		
*male (vs. female)*	0.112 (−0.731–0.954)	0.796
**Stenosis**		
***(yes vs. no)***	**2.401 (1.263**–**3.538)**	**<0.001**
**Q**_**a**_ **(mL/min)**	–**0.002 (**–**0.002 to** –**0.001)**	**<0.001**
**Q**_**x**_ **(mL/min/RI**)	–**0.001 (**–**0.002 to** –**0.001)**	**<0.001**
**RI**	**7.76 (2.77**–**12.76)**	**0.002**
Time from AVF creation, *months*	0.016 (−0.016–0.048)	0.332
**AVF dysfunction** ***(yes vs. no)***	**1.44 (0.55**–**2.33)**	**0.001**
Type of AVF	0.135 (−0.309–0.579)	0.553
CVD, %	0.690 (−0.195–0.1575)	0.127
**Systolic BP**, ***mmHg***	–**0.024 (–0.047 to –0.001)**	**0.040**
Diastolic BP, *mmHg*	−0.023 (−0.074–0.027)	0.356
Calcium, *mg/dL*	0.009 (−0.686–0.704)	0.980
Phosphorus, *mg/dL*	0.043 (−0.303–0.388)	0.809
Cholesterol, *mg/dL*	−0.001 (−0.012–0.009)	0.788
Hemoglobin, *g/dL*	−0.353 (−0.885–0.179)	0.193

*Bold values are statistical significant*.

**Table 3:** Multivariable Cox models on the risk of thrombosis.

	**Model with Q**_****a****_			**Model with Q**_****x****_		
	**HR**	**95% CI**	***P***	**HR**	**95% CI**	***P***
Systolic blood pressure, *mmHg*	0.97	0.95–0.99	0.016	0.98	0.95–0.99	0.024
AVF dysfunction, *yes vs. no*	2.39	1.15–4.96	0.019	2.35	1.13–4.86	0.022
Qa or Q_x_	0.99	0.98–0.99	<0.001	0.99	0.98–0.99	<0.001
*c*-Index	**0.766**			**0.778**		

*Bold values are statistical significant*.

C index of model with Q_x_ was higher than model with Q_a_, with the difference being statistically significant after applying the bootstrap method (0.012; 95%CI 0.004–0.019). This indicates a better discrimination ability of the model with Q_x_ as compared to that with Q_a_ on thrombosis occurrence.

## Discussion

In the present study, we have demonstrated the potential usefulness of Q_x_ as a new surrogate ultrasound marker to detect stenosis and predict thrombosis episodes in haemodialysis AVF. Q_x_ combines information from Q_a_ and RI, two other surrogate markers that have already been validated in literature and extensively used in daily practice. Yet, in our study, the Q_a_/RI ratio showed an improved diagnostic performance with respect to the single parameters considered alone. This finding may be of high clinical relevance as it may help overcoming the accuracy limitations that characterizes Q_a_ and RI, often leading to a misleading interpretation of AVF patency. In fact, lower RI values are not infrequently found in spite of set lumen narrowing in outflow vein, while a high flow rate is often maintained in early stenosis with lumen reduction (10, 19).

Different guidelines suggest variable criteria to approach AVF stenosis. In particular, it is still highly debated whether an immediate revascularization should be preferred over a “*wait and see*” strategy in order to prevent thrombosis occurrence (8, 20, 21).

The main aim of our investigation was to explore the validity of a new DUS parameter for critical stenosis to overcome hurdles that usually do not allow early intervention.

Such a tool could help in the decision process for a timely corrective procedure, like angioplasty, in order to maintain AVF patency for a longer time (22). Implications for health-care systems would be of utmost importance as vascular access failure remains a major cause of mortality and hospitalizations in the uremic population (23) with a consequent high economic burden (24).

In addition, stratifying stenotic lesions according to the risk of thrombosis remains a demanding priority. Several studies quantified the increased risk of infection and cardiovascular events after AVF failure (25) while other studies assessed the impact of conversion from native AVF to a catheter on mortality (26–29)_._ Recently the AURORA trial (30) displayed a strong association between AVF thrombosis and worsen patients' survival, emphasizing the impact of cardiovascular risk in the uremic population. The high mortality rate after AVF thrombosis was attributed to ionic imbalance related to the inability to perform a regular dialysis session, difficulties associated with central catheter placement as well as the revascularization procedure itself. In this respect, there is extensive agreement in current literature about the need of developing easy and validated tools for a better stratification of the thrombotic risk of AVF. Shintaku et al. found flow rate and resistance index immediately after AVF creation to be better predictors of early access failure and AVF maturation as compared to lumen diameter measurement. In fact, these two parameters may reveal functional changes preluding to morphological alterations leading to thrombosis (31).

According to our findings, Q_x_ could represent a possible way to improve even the detection accuracy of a single Q_a_ measurement, simply by performing a RI-based “normalization.” This was confirmed by the robust results obtained at Cox multivariate analyses in which a model including Q_x_ predicted the thrombotic event independently from other confounders such as age, sex and type of AVF and in a stronger manner than Q_a_.

The main strength of our study was the ample cohort and the long follow-up after the baseline assessment that allowed us to catch an adequate number of thrombotic events and, hence, to perform reliable multivariate adjustments and elaborated comparison of models. In addition, despite multicentre, the study population resulted quite homogeneous and the DUS procedures were highly reproducible across the different centers involved. The main limitation is probably represented by the lack of an angiographic exam as reference to confirm steno-thrombotic events. Digital subtraction angiography is nowadays recognized as the gold standard exam to characterize stenotic vascular lesions. However, the procedure is invasive, time- and cost-consuming, and the nephrotoxic radiopaque contrast medium injected to map vessels is not free from possible harmful effects.

Nevertheless, patients have been carefully checked and examined for a long list of possible clinical and DUS signs of stenosis/thrombosis and findings have been confirmed by a double check. Despite this, however, due to the intrinsic aim of the study and the overall characteristics of the cohort, the possible influence of selection bias on results cannot be fully ruled out.

In conclusion, Q_x_ has been found to be a more precise way than a simple Q_a_ or RI measurement for the rapid identification of a stenotic AVF and for stratifying the risk of a following thrombosis. Future, larger studies are needed to validate this parameter and to confirm whether such a simple measurement may really improve patterns of clinical practice by driving early re-vascularization or angioplasty before an overt thrombosis occurs.

## Data Availability Statement

The raw data supporting the conclusions of this article will be made available by the authors, without undue reservation.

## Ethics Statement

The studies involving human participants were reviewed and approved by Comitato Etico Calabria Centrale. The patients/participants provided their written informed consent to participate in this study.

## Author Contributions

AC and GC designed the study. DB and MP analyzed the data. GC had primary responsibility for the collection, analysis, and interpretation of the data and the final content. All authors contributed to drafting and writing the manuscript, read, and approved the final manuscript.

## Conflict of Interest

The authors declare that the research was conducted in the absence of any commercial or financial relationships that could be construed as a potential conflict of interest.
